# An analysis of freshmen’s knowledge, attitudes, and practices regarding cardiopulmonary resuscitation and the influencing factors at a university in Wuhu City: a cross-sectional study

**DOI:** 10.3389/fpubh.2025.1580600

**Published:** 2025-08-08

**Authors:** Zhengxia Yang, Yan Wang, Aoqi Dong, Xin Yang, Liqun Zhu, Shaoyong Ma, Yingying Wang

**Affiliations:** ^1^Department of Nursing, Yijishan Hospital of Wannan Medical College, Wuhu, China; ^2^Department of Nursing, Affiliated Hospital of Jiangsu University, Zhenjiang, China; ^3^School of Nursing, Wannan Medical College, Wuhu, China

**Keywords:** freshmen, cardiopulmonary resuscitation, knowledge, attitudes, practices

## Abstract

**Objective:**

The study aimed to investigate the current status of cardiopulmonary resuscitation (CPR) knowledge, attitudes, and practices (KAP) among freshmen at a college in Wuhu City. It also sought to identify the factors that influence these aspects and provide insights for enhancing CPR KAP among students.

**Methods:**

A convenience sampling method was used to select 1,550 college students from a university in Wuhu, Anhui Province, in 2024. A comprehensive general information questionnaire was developed following a review of databases, such as China Knowledge Infrastructure and PubMed. The CPR KAP scale, along with the General Self-Efficacy Scale and the eHealth Literacy Scale (eHEALS), were utilized for the web-based survey conducted through Questionnaire Star. Descriptive and quantitative methods were employed for statistical analysis.

**Results:**

Out of the 1,600 questionnaires collected, 50 responses were deemed invalid and subsequently excluded, resulting in 1,550 valid questionnaires and a validity rate of 96.7%. Consequently, the study included 1,550 participants, comprising 459 men and 1,091 women. The overall CPR KAP score for freshmen was 62.55 ± 13.74, with component scores for KAP being 31.17 ± 9.26, 24.56 ± 4.97, and 6.83 ± 5.51, respectively. Comparisons of scores across variables, such as gender, age, domicile location, self-perceived health status, parental education levels, first-aid training experience, presence of medical professionals in the family, experience with emergencies such as cardiac arrest, CPR training in previous curricula, and family health status, revealed statistically significant differences (*p* < 0.05). Pearson correlation analysis demonstrated a positive correlation between CPR KAP scores and both eHEALS (*r* = 0.358) and General Self-Efficacy Scale scores (*r* = 0.303), with both correlations being highly significant (*p* < 0.001). Multiple linear stepwise regression analysis identified eHEALS, first-aid training experience, inclusion of CPR training in the curriculum, gender, general self-efficacy, age, and domicile location as the primary factors influencing CPR KAP scores among freshmen.

**Conclusion:**

To enhance freshmen’s CPR KAP, future interventions should prioritize improving their general self-efficacy and eHEALS. Evidence suggests that strengthening self-efficacy can significantly enhance students’ confidence in performing CPR during emergencies. Furthermore, fostering eHEALS equips freshmen with the skills to comprehend and utilize health-related information effectively, which, in turn, promotes more positive attitudes and behaviors toward CPR adoption and practice. By focusing on these key areas, educational strategies can be developed to strengthen CPR preparedness among freshmen, ensuring that they are better equipped to respond in life-threatening situations.

## 1 Introduction

Cardiac arrest (CA) is a critical medical emergency that may result in fatality if not addressed promptly (1). Each year, approximately 2.5 million people in China die from sudden cardiac arrest, making it the leading cause of death globally (2). In the United States, nearly 500,000 people die from sudden cardiac arrest annually (3). More than 70% of sudden cardiac arrests occur outside of hospitals each year (4). Out-of-hospital cardiac arrest (OHCA), occurring outside hospital settings, poses a sudden and severe threat to human health (1). As a significant contributor to out-of-hospital mortality, OHCA has emerged as a major public health concern globally (5). The global incidence of OHCA varies from 40.8 to 100.2 cases per 100,000 person-years (6), affecting up to 5 million individuals annually worldwide (7). In China, the incidence stands at 97.1 per 100,000 person-years, marking an increase over the past decade (5). However, the survival rate of OHCA patients is less than 1% (8). Cardiopulmonary resuscitation (CPR), a fundamental life-support technique, is vital for managing cardiac arrests caused by diverse factors (9). Initiating bystander CPR early significantly enhances survival rates in OHCA incidents (10). Research indicates that performing CPR within the “golden 4 min” can boost survival chances by up to 50% (11). In Denmark, the implementation of compulsory CPR education in schools has resulted in a national bystander CPR prevalence rate of 41% and a more than threefold increase in survival rates (12). Bystander CPR, performed by laypersons, greatly improves survival prospects if commenced within 4–6 min following cardiac arrest (1). The American Heart Association (AHA) points out that immediate application of CPR can double or triple the survival chances of people who have experienced OHCA (13). The survival rate of patients who received bystander CPR is 2.6 times that of those who did not receive CPR (7). The survival rate of OHCA patients in Japan increased from 3.3 to 7.2%, due to a rise in the rate of bystander CPR from 38.6 to 50.9% (14). Although the rate of bystander CPR in several large and medium-sized cities in China has improved compared to before, the rate of bystander CPR and the survival rate of OHCA are only 4.5% (9). The rate of CPR training is between 5.74 and 25.6%, which is far lower than that of other countries (15). In Australia, 68% of respondents have received some form of CPR training (16). In Norway, 89% of middle school students have participated in CPR training (17). In Japan, 70% of the population has learned CPR, and 30% have learned it more than twice (18). Therefore, to increase the rate of bystander CPR, the government encourages large-scale first aid training, aiming to increase the proportion of people holding first aid training certificates to at least 3% by 2030 (19).

College students, with their advanced cultural literacy and theoretical knowledge, play a crucial role in disseminating CPR awareness and skills, which is crucial for enhancing first-aid capabilities and advancing public health (9). Moreover, training remains an effective method to improve the cardiopulmonary resuscitation skills of bystanders (20). At present, the majority of countries are conducting research on the knowledge, attitudes, and practices (KAP) of CPR among college students, as well as their training in other aspects of CPR. Compared to different occupations, college students show a higher willingness to learn CPR (21). Meanwhile, conducting cardiopulmonary resuscitation training for college students on campus has the advantages of fixed venues, continuity, systematic approach, and convenient assessment, which can effectively improve students’ mastery of CPR knowledge and skills among students (22). There are also some studies in China on the current situation of CPR knowledge, attitude, and practice among freshmen; however, most of these studies are conducted in specific areas. Currently, there is no study on the overall situation of KAP among freshmen in Wuhu City. This study focuses on freshmen as survey subjects, aiming to evaluate the current level of CPR KAP among college students in Wuhu City. It also aims to identify the factors influencing these aspects and encourages freshmen to actively learn the knowledge and skills of cardiopulmonary resuscitation to address their lack of medical knowledge. The results are expected to offer valuable data and scientific evidence to inform the design and implementation of first-aid education and training programs tailored for college students.

## 2 Methods

### 2.1 Study design

Between August 2024 to December 2024, 1,550 college students were selected as samples from a university in Wuhu City, Anhui Province, using a convenience sampling method. After reviewing databases such as China National Knowledge Infrastructure and PubMed, a comprehensive general information questionnaire was developed, and the CPR KAP scale, General Self-Efficacy Scale, and eHEALS scale were adopted. A web-based survey was conducted through the Wenjuanxing platform. *t*-tests, analysis of variance (ANOVA), and multiple linear stepwise regression analysis were performed to analyze the influencing factors of college freshmen’s KAP regarding CPR.

### 2.2 Participants

Inclusion criteria: Full-time undergraduate students who voluntarily participated in this study. Exclusion criteria: Incomplete questionnaire responses, inconsistent answers, or obvious contradictions between answers. A total of 1,550 university students from a certain university in Wuhu City, Anhui Province, participated in the online survey conducted via Wenjuanxing in 2024.

### 2.3 Ethical approval

This research has been approved by the Biomedical Ethics Committee of Wannan Medical College. All procedures comply with the requirements of the “Ethical Review Measures for Biomedical Research Involving Humans.” A data usage agreement has been signed, clearly stating that the data can only be used for this research. Sharing requires secondary desensitization and approval by the ethics committee. Send the research introduction email (including a summary of the approval letter from the ethics committee) through the educational administration system of the university, clearly stating the research purpose, process, risks, and benefits. Before data collection, surveyors who have completed ethical training will provide face-to-face explanations, emphasizing the principle of voluntary participation and ensuring that anyone can withdraw at any time without penalty. Data anonymization processing measures, commitment that all data collection tools have passed the pre-review of the ethics committee, and the rights and interests of participants take priority over the research goals. During the research process, no instances of informed consent withdrawal or psychological intervention occurred, and the representativeness of the final sample was not affected by ethical procedures. The informed consent rate was 100%, and there were no cases of forced participation, which was in full compliance with the Declaration of Helsinki.

### 2.4 Instrument

The samples for convenience sampling were all drawn from the freshmen of a university in Anhui Province. During military training, the questionnaires were filled collectively and supervised by instructors to enhance the efficiency and recovery rate of the questionnaires. This convenient sampling method will not have any impact on the responses of students of different genders. To achieve the research objectives, the CPR questionnaire for college students developed by D. Yeh et al. served as a reference for this study (23). The scale was utilized to generate the initial pool of items for the questionnaire, based on the 2015 American Heart Association Guidelines Update for Cardiopulmonary Resuscitation and Emergency Cardiovascular Care and relevant literature. This process was guided by nursing experts from the hospital’s emergency department and senior trainers from the Red Cross. The existing CPR questionnaire was tested on a group of college students, and adjustments were made based on interview results to enhance clarity and readability. Based on the feedback from participants in the pre-survey, further modifications were made to produce the final version of the questionnaire.

The final questionnaire consisted of 80 questions in five sections, including general information, CPR knowledge, CPR attitudes, willingness to practice CPR, eHEALS, and general self-efficacy. The CPR knowledge section contains 32 questions, of which questions 1–30 were multiple-choice. Each correct answer was awarded 1.5 points, and each incorrect answer was worth 0 points. Questions 31 and 32 are multiple-choice questions, with 2 points for all correct answers, 1 point for 60% correct answers, and 0 points for incorrect answers. The total score for this section is 49 points. The CPR attitudes section consisted of 10 questions, and the survey was conducted on a 4-point Likert scale, with “none” as 0, “usually none” as 1, “yes” as 2, and “very much so” 3 points. The CPR Behavior section consisted of 7 questions, with 0 being “not at all,” 1 being “not usually,” 2 being “sometimes,” and 3 being “often will.” The total score for this section was 21. The Cronbach’s alpha coefficient for the total scale was 0.880, and the coefficients for the three sections were 0.857, 0.902, and 0.915, respectively. The eHEALS scale consists of 8 items scored on a 5-point Likert scale (1 = strongly disagree, 5 = strongly agree). Total scores range from 8 to 40, with higher scores indicating stronger eHEALS. The Cronbach’s alpha coefficient for the scale was 0.960. The General Self-Efficacy Scale consists of 10 items rated on a four-point Likert scale (1 = “not at all true” to 4 = “completely true”). The higher the score, the higher the general self-efficacy. The Cronbach’s alpha coefficient for the scale in this study was 0.965, indicating excellent internal consistency, making it suitable for use by researchers.

The validity of the questionnaire was evaluated by two nursing experts and two senior Red Cross trainers, resulting in a content validity index of 0.88, which suggests that this questionnaire is reliable and valid. A convenience sample of 65 college students from one university completed the questionnaire during an initial survey. The same survey was administered 1 week later, yielding 48 compatible responses and demonstrating a re-test reliability of 0.84.

### 2.5 Data collection

To minimize measurement bias, all investigators underwent uniform training and were assessed for competency before the study. The questionnaires were distributed and collected using the Questionnaire Star platform, with internet protocol (IP) address restrictions to ensure on-campus access only. During military training, participants filled out the questionnaires collectively under the supervision of instructors to enhance both efficiency and recovery rate. Additionally, a dual mode of paper questionnaires and scanning codes was adopted to accommodate unstable network signals. The online questionnaire was open for 72 hours and automatically closed after the time limit. The term “Emergency Medical System (EMS)” was changed to “Wuhu 120 Emergency Medical Center.” Thirty freshmen were selected for cognitive interviews to clarify any ambiguous expressions, and two experts from the Wuhu Emergency Medical Center were invited to assess content validity, yielding a CVI score greater than 0.8. The system automatically excludes questionnaires completed in less than 3 minutes. The first page of the questionnaire clearly states the research purpose, anonymity, and data usage. Respondents must check “Agree” to proceed. Questionnaires with repeated responses or consistently selected options were flagged as invalid and excluded from analysis. These methodological safeguards provide a solid approach to minimizing bias, offering valuable reference for similar future studies.

### 2.6 Research variables

In this study, the KAP score for cardiopulmonary resuscitation for freshmen was used as the dependent variable. The independent variables include (whether there is experience in first aid training, whether the school incorporates cardiopulmonary resuscitation training into the curriculum, gender, age, and place of household registration), as well as the scores of the Electronic Health Literacy Scale and the General Self-Efficacy Scale.

### 2.7 Statistical analysis

Data analysis was conducted using IBM SPSS version 27.0 software. During the data entry stage, a double verification mechanism (entry by Researcher A + verification by Researcher B) was established, and variable attributes were strictly defined through the data view and variable view. Descriptive statistics were used for categorical variables such as gender. Independent sample *t*-tests (normal distribution) were conducted for continuous variables between the two groups, and univariate ANOVA analysis was used for multigroup comparisons. Pearson correlation analysis was used to measure the correlation between the scale scores. Accuracy calculation: Total score/number of items × 100%. Stepwise multiple linear regression was adopted, and R^2^ was adjusted. A difference was considered statistically significant when *p* < 0.05. Five emergency medicine experts were invited to score the item correlation. The items with I-CVI ≥ 0.78 were retained, and the S-CVI/UA of the entire scale was 0.89. Exploratory factor analysis (EFA): KMO = 0.84, Bartlett’s sphericity test *p* < 0.001, five common factors were extracted (cumulative variance explanation rate 67.2%), and the load of each item was >0.5.

## 3 Results

### 3.1 Participant sociodemographic information

A total of 1,600 questionnaires were collected, of which 50 were deemed invalid, resulting in 1,550 valid responses and a validity rate of 96.7%. The study included 1,550 participants, comprising 459 men (29.6%) and 1,091 women (70.4%). The age distribution of participants was as follows: 954 individuals were aged 18–19 (61.5%), 477 individuals were aged 19–<20 (30.8%), 100 individuals were aged 20–21 (6.5%), 14 individuals were aged 21: <22 (0.9%), and 5 individuals were aged 22–23 (0.3%).

### 3.2 Comparison of CPR KAP scores among different freshmen

A comparison of CPR KAP scores among freshmen revealed statistically significant differences (*p* < 0.05) based on various factors. These factors included gender, age, domicile, self-perceived health status, maternal and paternal education levels, first aid training experience, having relatives in the medical field, witnessing or personally experiencing emergencies such as cardiac arrests, prior inclusion of CPR training in school curricula, and family health status (Table 1).

**Table 1:** A comparison of CPR knowledge and practice scores among freshmen, considering their general information (*n* = 1,550).

Items	Number [*n* (%)]	score (^−^*x* ± s)	*T*/*F*	*p*
Gender			−4.657	<0.001
Men	459 (29.6)	60.06 ± 15.04		
Women	1,091 (70.4)	63.60 ± 13.02		
Age			3.872	0.004
18–19	954 (61.5)	63.51 ± 13.41		
19–20	477 (30.8)	61.46 ± 13.75		
20–21	100 (6.5)	59.02 ± 15.01		
21–22	14 (0.9)	62.65 ± 15.22		
22–23	5 (0.3)	56.00 ± 26.81		
Place of residence			9.999	<0.001
Countryside	1,228 (79.2)	61.79 ± 13.67		
Town	142 (9.2)	64.36 ± 14.61		
City	180 (11.6)	66.32 ± 12.80		
Monthly cost of living			1.671	0.154
<1,000	284 (18.3)	61.44 ± 14.14		
1,000 ~ <2,000	1,169 (75.4)	62.66 ± 13.69		
2,000 ~ <3,000	84 (5.4)	64.42 ± 12.37		
3,000 ~ <4,000	9 (0.6)	69.61 ± 15.86		
>4,000	4 (0.3)	56.38 ± 18.87		
Conscious health condition			9.109	<0.001
Very poor	14 (0.9)	67.21 ± 17.20		
Poor	57 (3.7)	56.19 ± 14.08		
General	786 (50.7)	61.20 ± 13.42		
Good	481 (31.0)	64.40 ± 12.68		
Very good	212 (13.7)	64.77 ± 15.72		
Mother’s education level			4.682	<0.001
Primary school or below	389 (25.1)	61.13 ± 13.86		
Primary schoolgraduate	339 (21.9)	61.37 ± 13.99		
Junior high school	550 (35.5)	62.90 ± 13.12		
Senior high school or vocational school	196 (12.6)	65.27 ± 13.50		
Associate degree or above	76 (4.9)	65.64 ± 15.54		
Father’s education level			4.084	0.003
Primary school or below	175 (11.3)	60.31 ± 15.46		
Primary school graduate	264 (17.0)	61.66 ± 12.27		
Junior high school	735 (47.4)	62.45 ± 13.38		
Senior high school or vocational school	278 (17.9)	63.65 ± 14.59		
Associate degree or above	98 (6.3)	66.62 ± 13.52		
Primary Caregiver			0.760	0.468
Parents	1,311 (84.6)	62.63 ± 13.89		
Grandparents	162 (10.4)	61.46 ± 11.85		
Other	77 (5.0)	63.60 ± 14.81		
First aid training experience			14.497	<0.001
Yes	489 (31.5)	69.55 ± 10.89		
No	1,061 (68.5)	59.33 ± 13.72		
Relative who is a paramedic			3.714	<0.001
No	294 (19.0)	65.22 ± 13.32		
No	1,256 (81.0)	61.93 ± 13.77		
Witnessed or experienced an emergency such as cardiac arrest?			2.757	0.006
Yes	130 (8.4)	65.73 ± 14.67		
No	1,420 (91.6)	62.26 ± 13.62		
Has CPR training been included in the curriculum of previous schools?			98.316	<0.001
Yes	729 (47.0)	67.43 ± 12.44		
No	258 (16.6)	59.12 ± 13.87		
Unknown	563 (36.4)	57.82 ± 13.17		
Health status of family members			10.567	<0.001
Very poor	20 (1.3)	65.70 ± 11.22		
Poor	97 (6.3)	58.94 ± 13.40		
General	710 (45.8)	60.76 ± 13.92		
Good	548 (35.4)	64.16 ± 12.85		
Very good	175 (11.3)	66.43 ± 14.66		

### 3.3 Correlation analysis of freshmen CPR KAP with eHEALS and general self-efficacy

Pearson correlation analysis demonstrated a positive correlation between freshmen’s CPR KAP scores and their eHEALS scores (*r* = 0.358, *p* < 0.001) as well as their General Self-Efficacy Scale scores (*r* = 0.303, *p* < 0.001). Both correlations were statistically significant, indicating that higher eHEALS and general self-efficacy are associated with better CPR KAP scores among freshmen (Table 2).

**Table 2:** Correlation analysis between freshmen CPR KAP scores and eHEALS and General Self-Efficacy Scale scores.

Scale	Correlation (r)	Minimum score	Maximum score	Score (^−^*x* ± *s*)	Total score
eHEALS	0.358***	8	40	32.28 ± 5.86	40
Self-Efficacy scale	0.303***	10	40	25.72 ± 7.28	40

***p < 0.001.

### 3.4 Multiple linear stepwise regression analysis of factors influencing freshmen’s CPR KAP

Multiple linear stepwise regression analysis was conducted with the total CPR KAP score of freshmen as the dependent variable. The study incorporated variables that demonstrated statistically significant differences in the univariate analysis, along with scores from the eHEALS and the General Self-Efficacy Scale. The specific values assigned to these variables are detailed in Table 3. The results identified the following factors as significant influences on freshmen’s CPR KAP scores: eHealth Literacy, first-aid training experience, inclusion of CPR training in the school curriculum, gender, general self-efficacy, age, and domicile location, all with (*p* < 0.05). These factors collectively explained 26.5% of the total variance in CPR KAP scores (Table 4).

**Table 3:** The way of assigning the independent variables of the factors influencing the knowledge, belief, and behavior of CPR among freshmen students.

Independent variable	Assignment method
Gender	Male = 1; Female = 2
Age	18 ~ <19 = 1; 19 ~ <20 = 2; 20 ~ <21 = 3; 21 ~ <22 = 4; 22 ~ <23 = 5
Place of residence	countryside = 1; town = 2; city = 3
Conscious health status	Very poor = 1; poor = 2; general = 3; good = 4; very good = 5
Mother’s education level	Primary school or below = 1; Primary school graduate = 2; Junior high school = 3; Senior high school or vocational school = 4; Associate degree or above = 5
Father’s education level	Primary school or below = 1; Primary school graduate = 2; Junior high school = 3; Senior high school or vocational school = 4; Associate degree or above = 5
First aid training experience	YES = 1; NO = 2
Relatives are medical personnel	YES = 1; NO = 2
Whether witnessed or personally experienced an emergency such as cardiac arrest	YES = 1; NO = 2
Whether CPR training was included in the curriculum of previous schools	YES = 1; NO = 2; Unknow = 3
Health status of family members	Very poor = 1; poor = 2; general = 3; good = 4; very good = 5
General self-efficacy	Raw Scale Score
eHEALS	Raw Scale Score

**Table 4:** Multiple stepwise regression analysis of factors affecting KAP in freshmen CPR.

Independent variable	Partial regression coefficient (B)	Standard error (SE)	Standardized partial regression coefficient (beta)	*t*	*p*
Constant	70.206	8.743		8.030	<0.001
eHEALS	0.430	0.063	0.183	6.845	<0.001
Any first aid training experience	−6.443	0.682	−0.218	−9.441	<0.001
Whether the school has included CPR training in the curriculum	−3.005	0.347	−0.198	−8.657	<0.001
Gender	3.259	0.669	0.108	4.869	<0.001
General self-efficacy	0.253	0.050	0.134	5.015	<0.001
Age	−0.994	0.442	−0.050	−2.247	0.025
Place of residence	0.993	0.451	0.049	2.200	0.028

*R*^2^ = 0.268, adjusted *R*^2^ = 0.265, *F* = 80.745, *p*<0.001.

### 3.5 CPR knowledge score and correctness rate of current college students

The CPR knowledge scores and correctness rates of current college students are shown in Table 5.

**Table 5:** CRP knowledge score and correctness rate of current college students.

Items	Score	Correctness rate (%)
1Normal respiratory rate per min for adults	0.72 ± 0.75	740 (47.7)
2Normal adult heartbeat per min	1.19 ± 0.61	1,233 (79.5)
3Do you think a healthy person can suddenly go into cardiac arrest?	0.63 ± 0.74	654 (42.2)
4The most common reasons for cardiac arrest in life	1.32 ± 0.48	1,368 (88.3)
5The “golden time” of CPR	1.06 ± 0.68	1,092 (70.5)
6How long does it take to judge when someone’s heart stops?	0.71 ± 0.75	737 (47.5)
7How to judge whether the collapsed person is conscious or not?	1.05 ± 0.69	1,084 (69.9)
8How to judge whether the injured person’s heartbeat stops?	1.23 ± 0.58	1,268 (81.8)
9How to judge whether the collapsed person has voluntary breathing?	1.09 ± 0.67	1,123 (72.5)
10 The casualty fell to the ground, to assess the safety of the environment or danger	1.37 ± 0.42	1,415 (91.3)
11 How much do you know about the emergency telephone number?	1.44 ± 0.28	1,492 (96.3)
12 When dialing 120, who should hang up first?	0.90 ± 0.73	933 (60.2)
13 When the heart stops, the person should take what kind of position?	1.22 ± 0.59	1,258 (81.2)
14 Cardiopulmonary resuscitation is the first blowing or first chest compressions	1.13 ± 0.65	1,164 (75.1)
15 Where should the casualty lie down during compression?	1.12 ± 0.66	1,152 (74.3)
16 The part of chest compressions	1.04 ± 0.69	1,070 (69.0)
17 It belongs to the correct compression	1.22 ± 0.58	1,263 (81.5)
18 Manner of chest compressions	0.74 ± 0.75	763 (49.2)
19 Depth of chest compressions	0.77 ± 0.75	795 (51.3)
20 How many times per min should chest compressions be performed?	0.53 ± 0.72	548 (35.4)
21 Can the palm of the hand leave the patient’s chest when doing chest compressions?	0.99 ± 0.71	1,023 (66.0)
22 Comparison of chest compression time and relaxation time	0.33 ± 0.62	338 (21.8)
23 Do you need to undress the casualty before blowing?	1.21 ± 0.59	1,252 (80.8)
24 Do you need to remove foreign objects from the mouth and nose before blowing?	1.26 ± 0.55	1,305 (84.2)
25 Do you need to pinch the nose when blowing?	1.11 ± 0.66	1,143 (73.7)
26 Should the mouth be wrapped when blowing?	0.93 ± 0.73	961 (62.0)
27 When blowing, should it be done quickly or slowly?	0.51 ± 0.71	531 (34.3)
28 How many times per min should you blow?	0.80 ± 0.75	823 (53.1)
29 Do you need to observe the patient’s chest or abdomen when blowing?	1.24 ± 0.56	1,285 (82.9)
30 When performing cardiopulmonary resuscitation, how do you match the number of compressions with the number of times you blow?	0.48 ± 0.70	498 (32.1)
31 How do you judge the success of CPR?	0.57 ± 0.83	578 (37.3)
32 When will you stop CPR?	1.26 ± 0.60	535 (34.3)

## 4 Discussion

### 4.1 Current status of freshmen CPR KAP scores

The total CPR KAP score of freshmen was (62.55 ± 13.74), with the knowledge, attitude, and behavior scores being (31.17 ± 9.26), (24.56 ± 4.97), and (6.83 ± 5.51), respectively. Among the knowledge-related questions, several had the correct answer rates below 40%, including: “How many times per min should chest compressions be performed,” “Chest compression time versus relaxation time,”” When blowing, should it be done quickly or slowly,” “How to match the number of compressions with the number of breaths blown during CPR,” “How to judge the success of CPR,” and “Under what circumstances should CPR be stopped.” These findings highlight a significant gap in freshmen’s understanding of CPR procedures. Specifically, the questions “How to judge the success of CPR” and “When to stop CPR” also had correct answer rates below 40%, further underscoring the need for improvement in CPR knowledge. To address these deficiencies, it is recommended that schools and relevant departments strengthen CPR education and training programs. Emphasis should be placed on increasing awareness and proficiency in this crucial life-saving skill. In 2015, the World Health Organization (WHO) approved the “Kids Save Lives” initiative that recommended schools worldwide should provide 2 h of CPR training each year. This recommendation was further supported by the 2023 statement of the International Resuscitation Liaison Committee (ILCOR). Different countries have adopted this suggestion to varying degrees. The Australian Resuscitation Council currently recommends a cardiopulmonary resuscitation refresher course once a year. Personalized CPR awareness plans need to be formulated for colleges and universities in Wuhu City based on international CPR awareness methods. A first aid course can be offered at universities in Wuhu City, allowing students to master the relevant skills and knowledge of CPR through a final assessment. A professional CPR publicity team should be established. Members of this team are required to have obtained CPR and first aid certificates (24, 25). Through personalized plans to popularize knowledge among students, the first aid ability of college students in Wuhu City can finally be improved. Furthermore, incorporating simulation drills and hands-on practical exercises is essential to enhance first aid knowledge and emergency response capabilities. By doing so, freshmen will be better equipped to respond effectively to emergencies, potentially saving lives in critical situations. Film and television works have a promotional effect on the popularization of CPR. According to social cognitive theory, when viewers observe certain behaviors in the characters they identify with on TV, they may induce changes in their own behaviors through concepts such as observational learning and increased self-efficacy (26).

### 4.2 Analysis of factors influencing the CPR KAP score among freshmen

#### 4.2.1 eHEALS

The findings of this study indicate that eHEALS significantly influences freshmen’s CPR KAP scores. The results of this study are in agreement with the results of the studies conducted by Vilela et al. (27). Freshmen with higher levels of eHEALS tend to achieve better CPR KAP scores. eHEALS is defined as an individual’s ability to access, understand, evaluate, and apply health information in an electronic environment to make informed health decisions (28). This underscores the importance of leveraging the internet, mobile devices, and other electronic tools to manage and promote health in the digital era. Students with high eHEALS are more proficient in utilizing online resources to acquire CPR-related knowledge and skills. They are better positioned to access the latest CPR guidelines, instructional videos, and simulation software, all of which enhance their understanding (29). Through effective e-health information dissemination, these freshmen can recognize the significance and urgency of CPR, fostering a positive attitude toward learning and practice. Additionally, individuals with high eHEALS are more likely to develop a correct understanding and positive attitude toward CPR. They may also prefer advanced technologies such as online simulations and virtual reality (VR) for hands-on CPR training. These technologies offer a more realistic and engaging training environment, thereby improving their practical skills and emergency response capabilities.

Colleges and universities should prioritize eHEALS education as a foundational component of their freshman curriculum. Equipping students with the skills to effectively access, evaluate, and apply health information from online sources will empower them to make informed decisions about their health. This includes fostering familiarity with reliable e-health platforms, mobile applications, and other digital resources that provide accurate and up-to-date health information. E-health platforms and social media can serve as valuable tools to widely disseminate CPR-related knowledge, raising awareness and enhancing CPR KAP scores. Institutions should leverage these digital tools to share instructional videos, infographics, and interactive content that promote a deeper understanding of CPR techniques and procedures. Furthermore, combining online simulation-based training with offline, hands-on practical sessions can create a comprehensive and engaging learning experience. Online simulations, including virtual reality and interactive software, can provide students with a safe and realistic environment to practice CPR techniques. Offline practical training, such as using mannequins and participating in real-time drills, can complement these digital experiences by allowing students to refine their skills and build confidence in physical application. By integrating both approaches, colleges and universities can offer diverse opportunities for freshmen to improve their CPR proficiency, ensuring they are better prepared to respond effectively in real-life emergencies.

#### 4.2.2 Experience of first aid training

The study found that freshmen with prior first aid training had higher CPR KAP scores. First aid training typically includes foundational CPR theory, such as recognizing cardiac arrest, performing chest compressions and artificial respiration, and using an automated external defibrillator (AED) (30). This training emphasizes the critical importance and urgency of first aid in emergencies. Through first aid training, students come to appreciate the life-saving potential of CPR, fostering a positive attitude toward continued learning and practice. These programs often incorporate simulation exercises and hands-on sessions, enabling students to apply theoretical knowledge in practical settings. Repeated practice helps students master CPR techniques and enhances their ability to respond to emergencies. To further improve CPR skills and emergency preparedness, colleges, universities, and relevant departments should bolster first aid training programs for new students. Regular activities, such as first aid competitions and simulation drills, can increase students’ interest and enthusiasm for learning. These engaging and interactive approaches not only make learning more enjoyable but also contribute to higher CPR KAP scores.

#### 4.2.3 Inclusion of CPR training in the curriculum at previous schools

The study indicates that students achieve higher CPR KAP scores when CPR training is incorporated into their previous school curricula. As the most fundamental form of first aid, CPR is critical for survival when administered promptly by the first witness (31). Early administration of CPR significantly increases the chances of survival, with success rates potentially reaching up to 90% (32). Incorporating CPR training into the curriculum provides students with a structured opportunity to learn and master essential CPR knowledge systematically. This includes recognizing cardiac arrest, performing chest compressions, and executing artificial respiration techniques. As a result, students’ knowledge scores improve directly. Moreover, students gain an understanding of CPR’s vital role in saving lives, which fosters a positive attitude toward learning and practice. This positive mindset equips them to respond more calmly and decisively during emergencies, thereby improving their attitude scores. CPR training typically involves simulation exercises and practical sessions (33). These experiences enable students to translate theoretical knowledge into practical skills, thereby enhancing their ability to manage real-life emergencies. Through repeated practice, students can significantly improve their practical skills, which enhances their practice scores. To maximize the benefits of CPR training, schools should incorporate it into their regular curriculum. This approach not only enhances students’ CPR proficiency but also prepares them to respond effectively in critical situations, ultimately contributing to better health outcomes in the community.

#### 4.2.4 Gender

The study revealed that women generally have higher CPR KAP scores compared to men. Women are often more health-conscious and tend to adopt healthier lifestyles, which may drive them to prioritize health education and first aid knowledge (34). This greater emphasis on health awareness contributes to a more positive attitude toward CPR and enhances their ability to acquire CPR-related knowledge. The difference in CPR KAP scores between genders highlights the need for gender-sensitive approaches to first aid training programs. Tailoring training methods to the unique characteristics, preferences, and needs of different genders can help optimize learning outcomes. For instance, men, who may be less inclined to seek out or participate in health education initiatives, could benefit from targeted strategies to increase engagement and motivation. On the other hand, women’s natural inclination toward health education can be further supported by providing leadership opportunities in first aid training programs. By incorporating these gender-sensitive training methods, institutions can ensure more inclusive and effective first aid education, ultimately improving CPR proficiency for all students regardless of gender.

#### 4.2.5 General self-efficacy

The study suggests that new students with high general self-efficacy tend to achieve higher CPR KAP scores. General self-efficacy refers to an individual’s overall confidence in their ability to handle tasks or challenges across various environments, which helps buffer the negative psychological effects of stress. Freshmen with high self-efficacy are more likely to view CPR as an important and useful skill, maintaining a positive and optimistic attitude toward learning it (35). They are more willing to help others in need and trust their ability to make sound judgments and take appropriate action. Consequently, they demonstrate higher practice scores. To foster self-efficacy, educational programs can incorporate confidence-building activities and provide positive reinforcement during CPR training. Encouraging students to set achievable goals and offering constructive feedback can further enhance their self-efficacy, leading to improved CPR proficiency.

#### 4.2.6 Age

Age is another significant factor affecting CPR KAP scores, with older freshmen generally scoring lower compared to their younger counterparts. Younger students often display greater receptivity to new knowledge, including CPR-related skills, and maintain a more optimistic attitude toward the importance of these skills. Educators can support older freshmen in improving their CPR KAP scores by adopting individualized teaching approaches, providing increased opportunities for practice, and emphasizing health education. Tailored strategies such as personalized feedback, mentorship, and adaptive learning techniques can help older students overcome potential barriers and enhance their ability to handle emergencies effectively. By addressing age-related challenges through targeted interventions, educational institutions can ensure that all freshmen, regardless of age, are well-prepared and confident in their CPR skills.

#### 4.2.7 Place of residence

The study found that freshmen from urban and township areas generally achieved higher CPR KAP scores compared to their counterparts from rural areas. Urban and township regions typically benefit from superior educational resources, such as more CPR training courses, access to professional instructors, and advanced simulation equipment (36). These resources enable students from these areas to learn CPR more systematically and effectively, fostering positive attitudes and enhancing skills through practice.

In contrast, rural areas may face challenges such as insufficient policy support and limited institutional resources for promoting first aid knowledge and skills. This can hinder the learning and practice of CPR among students in these regions, contributing to lower KAP scores.

To bridge this gap, the government and communities should invest more in CPR education within rural areas. Increasing access to learning opportunities and practical training can help rural students develop the necessary skills and confidence in performing CPR. A collaborative effort involving government bodies, societal organizations, and students is crucial to strengthening first aid education and training across these underserved regions.

By addressing these disparities, we can ensure that all students, regardless of their place of residence, have the opportunity to become proficient in CPR, ultimately contributing to better emergency preparedness and health outcomes across the board.

### 4.3 Implication

First, schools should strengthen the popularization of CPR knowledge, especially the training of key skill components, such as the standard frequency of chest compressions and the timing of compressions and relaxation. By increasing the number of simulation drills and practical opportunities, students can be helped to better master the skills of CPR and their confidence in coping with emergencies. Secondly, training programs should be tailored according to the characteristics of different groups of people. For example, for students in rural areas, online learning platforms can be increased to provide them with convenient learning resources. Meanwhile, considering the advantages of female students in CPR learning, future training can be designed with more attractive learning modes to motivate male students to participate more actively. Engaging with the local Red Cross Society allows for the acquisition of standardized teaching materials, instructor certification, provincial Red Cross project support, and financial support from local enterprises (such as medical device companies) or government health project funding. In addition, CPR will be included in the freshman enrollment education/elective course credits to improve the popularization rate of students’ CPR knowledge, as well as cultivate the awareness of college students to perform first aid when encountering sudden cardiac arrest patients, and finally through the implementation of student clubs (such as first aid clubs, youth associations), to help college students proficient in the use of CPR first aid. The student union can also organize college students to publicize CPR knowledge.

### 4.4 Core implication

To enhance the CPR skills of freshmen, it is necessary to identify their key weak points, seize the “window period” of enrollment, and conduct targeted interventions through high-quality simulation exercises and emergency rescue publicity campaigns. This is not only crucial for improving CPR education in universities, but also constitutes a universal framework for enhancing emergency preparedness education and health literacy. Therefore, regarding CPR training as a key measure to improve the health literacy and emergency response capabilities of freshmen is of great significance.

### 4.5 Strengths and limitations

This study focused on the freshmen of a certain university in Wuhu City and conducted an investigation targeting this specific group, filling the gap in the research on the KAP of CPR among college students. Freshmen, as representatives of the new forces in society, the research results can provide a scientific basis for the formulation of first aid education policies in colleges and universities. Furthermore, the research results directly reveal the necessity of incorporating CPR training courses into school education and provide a basis for differentiated training strategies for different genders, household registration, and age groups, which has high practical application value. This study also has certain limitations. First, this study employs a cross-sectional design, which can only reflect the correlation between variables and cannot determine the causal relationship. Relevant longitudinal studies can be conducted in the future to explore the causal relationship between them. Secondly, this study only selected freshmen from one university in Wuhu City. The sample size is limited, and there may be deviations in regions and types of institutions. In the future, it can be expanded to multiple regions, various types of universities, and students of different grades to enhance the universality and representativeness of the results. Thirdly, online questionnaires rely on self-reports from participants and may be affected by the social expectation effect (such as avoiding or embellishing responses to sensitive questions), resulting in a decrease in the authenticity of the data. Future research can adopt a combination of subjective and objective evaluation methods, such as the objective evaluation tool of KAP to assess students’ cardiopulmonary resuscitation skills taught by clinical teachers.

### 4.6 Recommendations

This study demonstrates that CPR training has a significant effect on improving college students’ first aid knowledge, attitudes, and behaviors. Notably, students with more extensive first aid training experience exhibit significantly better CPR knowledge and practice than those who have not received training. Based on this finding, the implementation of mandatory CPR training becomes one of the effective ways to improve college students’ first aid competence.

However, this study also identified some learning barriers, especially in the mastery of key skills such as “number of chest compressions per min” and “timing of compressions and relaxation,” in which many students had a low rate of correctness. Possible reasons for this include the lack of sufficient practice opportunities and simulation drills, which led to the students’ low confidence in the actual operation. In addition, some students’ understanding of CPR remains theoretical and lacks practical experience.

The mandatory inclusion of CPR training in the university curriculum as part of public health education is a topic worth exploring. However, this study found that first aid training significantly increased students’ KAP scores, more empirical research is needed to verify the long-term effects and feasibility of making this a mandatory course or incorporating it into public health programs. Future studies could further explore whether mandatory CPR training is effective in filling the gaps in CPR knowledge, attitudes, and behaviors among freshmen through comparisons between different regions and colleges. In addition, the effects of different training modes, such as online and offline combined training as well as virtual reality technology-assisted training, can be studied to assess their advantages in improving CPR skills. The findings of this study reveal that there are significant gaps in freshmen’s CPR knowledge and skills, especially in some specific operational skills. Therefore, future research should further explore the dilemmas and needs of freshmen in CPR learning and identify specific factors that affect their learning outcomes, such as psychological barriers, knowledge acceptance, and the availability of training resources. Based on these findings, educators can develop targeted teaching strategies to help freshmen overcome learning barriers effectively. To further promote CPR training, future research should also explore the scalability of incorporating CPR training into university curricula and public health programs. CPR training support could be provided to more schools through collaboration with public health departments and by combining existing educational resources. At the same time, consideration could be given to incorporating CPR training as part of university public health education programs, so that it is not limited to medical-related majors, but covers students from all majors. In addition, the promotion of CPR education should also be combined with social resources, using the power of all sectors of society, such as the Red Cross and other organizations, to jointly promote the popularization of first aid skills for all.

## 5 Conclusion

This study found that freshmen at a certain university in Wuhu exhibited a notable knowledge deficiency in cardiopulmonary resuscitation. The correct rate of students in the key operations of CPR was less than 40%, but their learning attitude was positive (with a score of 24.56/30). The training effect was remarkable. The total KAP score of students who received first aid training (69.55 points) was significantly higher than that of those who did not receive training (59.33 points). The disparity between urban and rural areas is prominent. The score of students with urban household registration (66.32 points) is higher than that of rural students (61.79 points), reflecting the uneven distribution of educational resources. Therefore, it is recommended that CPR training be included in the mandatory courses in colleges and universities, complemented by theoretical instruction and simulation assessment, to ensure that graduates possess the fundamental first aid skills. Meanwhile, modular teaching is designed for the weak links of knowledge, and digital tools such as VR and application (APP), are utilized to enhance the training efficiency. The gap in emergency rescue capabilities between urban and rural areas can also be narrowed through “university-community” collaboration (such as training for college students returning to their hometowns and mobile ambulances going to rural areas). This research is of great significance. For the first time, it focuses on the group of freshmen and clarifies the key directions for improvement in CPR education.

## Data Availability

The original contributions presented in the study are included in the article/supplementary material, further inquiries can be directed to the corresponding author.
